# Iatrogenic Femoral Arteriovenous Fistula with Double Connection between Femoral Artery and Vein Leading to High-Output Heart Failure Years after Mitral and Tricuspid Valve Replacement

**DOI:** 10.1155/2013/712089

**Published:** 2013-05-16

**Authors:** Arda Özyüksel, Rıza Doğan

**Affiliations:** ^1^Medipol Üniversitesi Kalp ve Damar Cerrahisi Bölümü, TEM Otoyolu Göztepe Çıkışı, No. 1, Bağcılar, 34214 İstanbul, Turkey; ^2^Department of Cardiovascular Surgery, Hacettepe University, Ankara, Turkey

## Abstract

Arteriovenous fistulas (AVFs) are potentially harmful but curable complications of diagnostic and interventional cardiac catheterizations. In this report, we present a case of iatrogenic AVF both from superficial and deep femoral arteries to common femoral vein leading to progressively developing severe right-sided heart failure in a patient who had two normally functioning mechanical valves at mitral and tricuspid positions. A 58-year-old woman who had a history of mitral and tricuspid valve replacement operations was admitted to our clinic with exertional dyspnea, palpitation, abdominal tenderness, and right-sided inguinal pain. Coronary angiography was performed via right femoral arterial access ten months ago. Doppler ultrasonography and computerized tomography revealed right superficial femoral artery to common femoral vein fistulisation. The patient was operated, and a double connection between the femoral artery and vein was encountered and treated successfully. Soon after the surgical procedure, clinical signs of right-sided heart failure ceased dramatically. The postoperative course was uneventful. Access site complications following interventional procedures represent significant problems. Sudden and progressive clinical deterioration in a patient especially with a history of diagnostic or therapeutic cardiovascular intervention may evocate the possibility of peripheral access site AVF formation.

## 1. Introduction

Diagnostic and interventional cardiac catheterizations, the most common site of arterial access being the femoral artery, have increased recently [1]. Arteriovenous fistulas (AVFs) are potentially harmful but curable complications of cardiac catheterization. Since these fistulas can cause congestive heart failure and may threaten limb circulation, prompt diagnosis and appropriate treatment are essential [2].

In this report, we present a case of iatrogenic AVF both from superficial and deep femoral arteries to common femoral vein leading to progressively developing severe right-sided heart failure in a patient who had two normally functioning mechanical valves at mitral and tricuspid positions. The patient had a medical history of diagnostic cardiac catheterization at the affected limb site. Surgical repair was successfully performed, and clinical signs and symptoms diminished dramatically afterwards. 

## 2. Case Presentation

A 58-year-old woman who had a history of mitral (14 years ago) and tricuspid (7 years ago) valve replacement operations was admitted to our clinic with exertional dyspnea, palpitation, abdominal tenderness, and right-sided inguinal pain. She had been followed up with irregular intervals. It had been more than a year since her last echocardiographic evaluation, and the mechanical valves were reported to be functioning normally at that time. In her medical history, there was an attempt for coronary angiography which was performed via right femoral arterial access ten months ago. Her coronary angiography was reported to be normal. Femoral cannulation was not performed at her cardiac operations.

Beginning four months ago, she had complaints of exertional dyspnea, palpitation, right-sided abdominal tenderness, and pain at the right groin area. On physical examination, any cardiac murmur was not noticed. She had hepatomegaly and mild peripheral pitting edema. Physical examination of lower extremity revealed continuous bruit on the right groin with palpable thrill. Lower extremity peripheral pulses were weakened at the right side. Branham's sign was present.

Laboratory evaluation did not reveal any abnormality but mildly elevated hepatic enzymes. Echocardiography was performed; mitral and tricuspid valves were reported to be functioning normally, and there was neither stenosis nor insufficiency. Left ventricular systolic and diastolic functions were normal. There was biatrial dilatation without any thrombus formation. 

**Figure 1:**
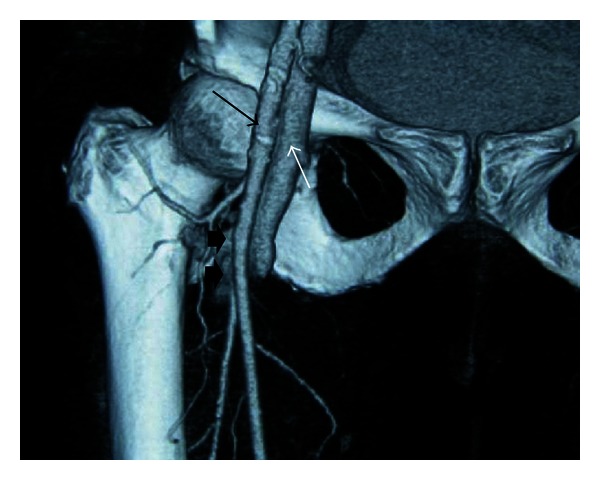
CT angiography examination of the patient. Contrast passage between the femoral artery and vein (arrow heads) is demonstrated. (Black arrow: common femoral artery, white arrow: common femoral vein).

With the provisional diagnosis of peripheral AVF leading to a new onset right-sided heart failure, Doppler ultrasonography was performed and revealed right superficial femoral artery to common femoral vein fistulisation. Contrast enhanced lower extremity computerized tomography was performed afterwards, which revealed the connection between superficial and deep femoral arteries to common femoral vein and the early phase contrast enhancement in iliac veins and inferior venae cavae (Figure 1).

**Figure 2:**
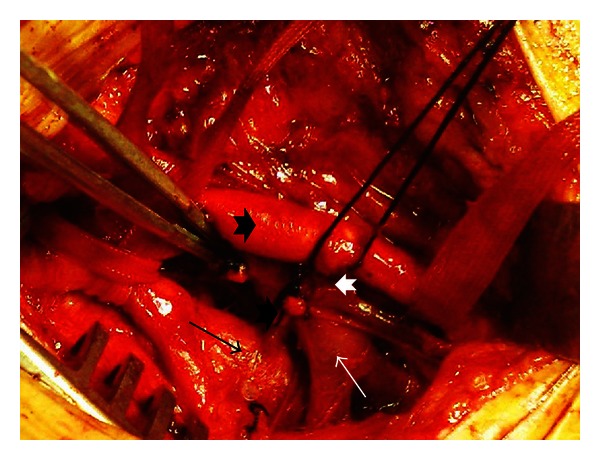
Two discrete arteriovenous fistulas are exposed; the first one (white arrow head on the left side) is between deep femoral artery (black arrow) and femoral vein (white arrow), and the other one (white arrow head on the right side) is between superficial femoral artery (black arrow head) and femoral vein.

The patient was operated under general anesthesia. Right vertical groin incision was performed. Superficial femoral artery (SFA) and deep femoral artery (DFA) were explored. A narrow-based AVF between DFA and common femoral vein (FV), 3 cm distal to DFA origin, was detected. A second connection with a broad-based neck was located between SFA and FV, at the fourth centimeter of SFA (Figure 2). Both were occluded temporarily, and the thrill ceased. The narrow-based AVF between DFA and FV was ligated and divided at arterial and venous ends. For the second AVF, SFA at the broad-based junction was resected. The venous site was sutured from within the artery. After resection of a ten millimeter segment of artery, SFA was repaired with end to end anastomosis. An autologous arterial wall patch was secured on half distance of the anastomosis. After the procedure thrill was totally ceased, both SFA and DFA pulses were present.

## 3. Results

Soon after the surgical procedure, clinical signs of right-sided heart failure ceased dramatically. The postoperative course was uneventful, and the patient was discharged five days after the operation.

## 4. Discussion

Access site complications following interventional procedures represent significant problems and also increase morbidity and mortality [3, 4]. Fistulas resulting from femoral artery punctures are usually small in size, and they can close spontaneously in a couple of months. However, if they persist, arterial and venous walls dilate, become weaker, and may lead to an increased cardiac output [5]. Later on the course, congestive heart failure may result.

Diagnosis of an AVF is mostly based on medical history and physical findings. The most common clinical features are swelling, thrill, bruit, and pulse deficit in the affected limb. Enlarging left-to-right shunt may lead to chronic volume overload and cause congestive heart failure. Our patient was admitted to our clinic with progressive symptoms of heart failure. Since she had a history of mitral and tricuspid valve replacement, mechanical valve dysfunction was focused on first. Physical examination revealed thrill on the right femoral artery and weakened arterial pulses on the right lower extremity, which then led us to consider AVF formation.

Since arterial puncture of superficial and deep femoral arteries is associated with arterial complications, proper selection of puncture site in order to gain access to common femoral artery is critically important. In our case, fistulas were arising both from superficial and deep femoral arteries indicating that the probable puncture site was below the level of common femoral artery.

Branham's sign, which is a decrease in pulse and increase in blood pressure that follows the sudden occlusion of an AVF, represents a hemodynamically significant AVF in the circulation and was present at the patient's physical examination.

As a result, sudden and progressive clinical deterioration in a patient especially with a history of diagnostic or therapeutic cardiovascular intervention may evocate the possibility of peripheral access site AVF formation. Moreover, arterial or venous access must carefully be gained in patients with a history of cardiac disease or surgery, since cardiac catheterizations may be needed in the follow-up course of such patients; peripheral arterial and venous access sites may be traumatized repeatedly. Femoral vessels exposed for peripheral cannulation at the time of cardiac surgery may have increased risk for AVF development.
